# Microbial Composition, Antibiotic Sensitivity Patterns, and Contributing Factors Among Children With Tonsillitis in Hawassa Town, Sidama, Ethiopia

**DOI:** 10.1155/ijm/6366378

**Published:** 2025-04-16

**Authors:** Tebiku Daniel Tirago, Mesfin Worku, Tadesse Menjetta Nima, Moges Desta Ormago

**Affiliations:** School of Medical Laboratory Science, College of Medicine and Health Sciences, Hawassa University, Hawassa, Ethiopia

**Keywords:** antibacterial susceptibility pattern, Ethiopia, Hawassa, sore throat, tonsillitis

## Abstract

**Background:** Infections of the tonsils are very frequent among 5–14-year-old children due to poor immunity establishments and inflammation within the tonsils because of insufficient penetration of antibiotics into the tonsillar core. This study was aimed at determining the bacterial profile, antibacterial susceptibility pattern, and associated factors among children with tonsillitis from selected health facilities in Hawassa town, Sidama, Ethiopia.

**Methods:** An institution-based cross-sectional study was conducted using interviewer-administered, pretested questionnaires, and throat swab samples were collected from children with tonsillitis visiting selected health facilities in Hawassa town. A systematic random sampling technique was used to select the study units. Antimicrobial susceptibility testing was carried out by using the disc diffusion method according to criteria set by the Clinical Laboratory and Standard Institute 2020. Logistic regression evaluated factors related to the prevalence of culture-confirmed bacterial tonsillitis.

**Results:** The overall prevalence of bacterial tonsillitis among children (408) with tonsillitis among selected health facilities was 276/408 (67.6%) (95% CI: 63.0%, 72.0%). The dominant bacterial isolate was *Streptococcus pyogenes* (70) (25.4%), followed by *Streptococcus aureus* (59) (21.4%), *Staphylococcus epidermidis* (56) (20.3%), *Streptococcus pneumoniae* (35) (12.7%), and *Haemophilus influenzae* (27) (9.8%). *S. pyogenes* was resistant to cephalexin (45.7%), penicillin (44.3%), and ceftriaxone (42.9%). Higher odds of having bacterial tonsillitis were observed for children from low monthly income families (adjusted odds ratio (AOR) 2.31, 95% CI: 1.15–4.63), those with enlarged or tender glands (AOR 2.47, 95% CI: 1.57–3.88), and those with a history of recurrent tonsillitis (AOR 1.85, 95% CI: 1.18, 2.92).

**Conclusions:** Bacterial tonsillitis was prevalent in the study area. The isolates showed resistance to common antibiotics such as penicillin, chloramphenicol, clindamycin, ceftriaxone, and cephalexin. Therefore, culture and susceptibility tests are vital for appropriately managing bacterial tonsillitis.

## 1. Introduction

Tonsillitis remains a frequently occurring clinical problem, affecting children and adults and contributing to significant socioeconomic impact worldwide [1]. Tonsillitis caused by bacteria typically occurs in children aged 5–15 years, rare in those less than 2 years and above 15 years of age [2].

Tonsillitis is among the most common childhood diseases [3]. It has a significant impact on health status and quality of life as it causes significant morbidity, time lost for school or work, and treatment costs [2]. More than 750,000 tonsillectomies are performed annually in the United States, and tonsillitis is a common indication [4]. Peritonsillar abscess/quinsy, airway obstruction, poststreptococcal glomerulonephritis, and rheumatic fever are the most common complications of tonsillitis [5].

Much has been written about the causative agent of tonsillitis. While a single microbial species may cause tonsillitis, it has been reported that tonsillitis is a consequence of polymicrobial infection, and microorganisms other than Group A beta-hemolytic streptococcus (GABHS) may be the cause of tonsillitis [6, 7].

The inappropriate overuse of antimicrobial agents and empirical treatment can lead to the development of antibiotic resistance. Like other infectious diseases, the determination of the pathogenic agents is important in the antibiotic selection for the medical treatment of tonsillitis. Hence, information on tonsillitis and drug susceptibility patterns is very important for the proper selection and use of antibiotic agents. Therefore, this study was aimed at determining the bacterial profile, antimicrobial susceptibility pattern, and associated factors among children with tonsillitis in Hawassa town, Sidama, Ethiopia.

## 2. Materials and Methods

### 2.1. Study Area and Period

The study was conducted in selected health facilities (one hospital, two health centers, and 10 private clinics) in Hawassa City from March 1 to April 30, 2021. Hawassa is the capital city of the Sidama Regional State of Ethiopia. The city had a total population of 328,283. Hawassa City had three government and five private hospitals, 10 government health centers, 57 medium clinics, and seven specialized clinics, with a total of 82 healthcare facilities.

### 2.2. Study Design and Participants

The institution-based cross-sectional study was conducted in selected health facilities in Hawassa City. The source population was all children visiting healthcare facilities in Hawassa town, Sidama Region, Ethiopia, and children visiting the selected healthcare facilities in Hawassa town during the study period were the study population. A proportionally allocated sample size was obtained through a systematic random sampling technique from each facility.

The sample size was determined using a single proportion formula, considering the 95% confidence interval (CI) (*z* = 1.96), taking the prevalence (p) as 50% since a similar study was not found in the country and the margin of error (5%) (*d* = 0.05) with 10% contingency. The final sample size was 422.

### 2.3. Data Collection and Laboratory Processing

All relevant sociodemographic and clinical data were collected by trained nurses from participants'/children's guardians through face-to-face interviews at pediatric OPD using a semistructured questionnaire. The throat sample was collected from each participant using a sterile cotton swab and placed immediately in Amies Transport Medium and transported to Hawassa Public Health Microbiology Laboratory.

### 2.4. Cultivation and Identification of Isolates

The throat swabs were inoculated with blood agar (Oxoid, Hampshire, United Kingdom), MacConkey agar (Oxoid, Hampshire, United Kingdom), and chocolate agar (Oxoid, Hampshire, United Kingdom). The inoculated MacConkey and blood agar were incubated aerobically at 37°C for 24 h, whereas the inoculated chocolate agar was incubated using a 5%–10% CO_2_-generating candle jar at 37°C for 24 h. After 24 h incubation, the plates were examined for growth, and colonies were preliminarily characterized by colony morphology, gram stain, and hemolytic reactions on blood agar plates. Identification of bacteria down to species level was done by biochemical tests such as the catalase, coagulase, optochin (30 *μ*g) test, bacitracin (30 *μ*g) test, and novobiocin (5 *μ*g) test for Gram-positive bacteria identification. Oxidase, indole production, urease, citrate utilization, lysine decarboxylation, carbohydrate fermentation, gas production, H_2_S production, and motility were used for the identification of species of Gram-negative bacteria [8].

### 2.5. Antimicrobial Susceptibility Test (AST)

AST was performed for identified bacterial isolates by disc diffusion technique as recommended by the Clinical and Laboratory Standard Institute on Mueller–Hinton agar (Oxoid, Hampshire, United Kingdom) and 5% sheep blood supplemented Mueller–Hinton agar for fastidious bacterial isolates. Based on CLSI (2020) guidelines, the following antibiotics were used for this study: ceftriaxone (30 *μ*g), ciprofloxacin (5 *μ*g), penicillin (10 *μ*g), amoxicillin-clavulanate (20/10 *μ*g), ampicillin (10 *μ*g), vancomycin (30 *μ*g), tetracycline (30 *μ*g), erythromycin (15 *μ*g), azithromycin (15 *μ*g), chloramphenicol (30 *μ*g), and cephalexin (30 *μ*g). Finally, the result was reported as sensitive (S), intermediate (I), or resistant (R) by measuring the diameter of the zone of inhibition or hemolysis [9].

### 2.6. Quality Control

Before actual data collection, the quality of data was assured by pretesting questionnaires on 5% of the sample size before actual work to assess its clarity and to make amendments, and orientation was given to data collectors. The prepared culture media were checked for sterility by incubating 5% of the prepared media for 24 h and observing for the presence of any colony growth. In addition, the abilities of the prepared media to support the growth of organisms were checked by inoculating control strains. Standard reference strains of *Streptococcus pyogenes* (ATCC 19615), *Streptococcus agalactiae* (ATCC 13813), *Streptococcus pneumoniae* (ATCC 49619), *Haemophilus influenzae* (ATCC-49766), and *Escherichia coli* (ATCC 25922) were used during culture and ATS.

### 2.7. Data Processing and Analysis

All filled questionnaires for this study were checked visually, coded and entered into Excel, and then exported to SPSS Version 26 software (SPSS Inc., Chicago, IL, United States) for analysis. Bivariate logistic regression was used to determine factors of culture-confirmed bacterial tonsillitis. For those variables with a *p* value < 0.25 in the bivariate, the analysis was further entered into the multivariable logistic regression model. Associations between dependent and independent variables were assessed, and their strengths were described using odds ratios at 95% CIs. A statistically significant association was considered as *p* value < 0.05.

## 3. Result

### 3.1. Sociodemographic Characteristics

A total of 408 participants were enrolled in this study with a response rate of 96.68%. Of them, 233 (57.1%) were female. The mean age of patients was 9.4 ± 2.85. The majority of the study participants (347) (85.0%) were urban dwellers (Table 1).

### 3.2. Behavioral and Clinical Characteristics

Among 408 study participants, 226 (55.4%) had a history of recurrent tonsillitis more than three times in a year, 199 (48.8%) had a history of tonsillitis in the family, and 210 (51.5%) were children living in a clean environment. More than half of the respondents reported that 232 (56.9%) children ate spicy foods, and 202 (49.5%) children ate or drank cold food (Table 2).

Signs and symptoms of the children with tonsillitis at selected health facilities were mainly fever (353) (86.5%); white or yellow coating or patches on the tonsils (286) (70.1%); a scratchy, muffled, or throaty voice (285) (69.9%); sore throats (280) (68.6%); and difficult or painful swallowing (275) (67.4%) (Table 3).

### 3.3. Bacterial Profile and Associated Factors of Bacterial Tonsillitis

The overall p of bacterial tonsillitis among children with tonsillitis in this study was 276 (67.6%) (95% CI: 63%–72%). The p of Gram-positive bacteria (242) (59.3%) predominated over Gram-negative bacteria (34) (8.3%). In this study, *S*. *pyogenes* (70) (17.2%) was frequently isolated bacteria, followed by *Streptococcus aureus* (59) (14.5%), *Staphylococcus epidermidis* (56) (13.7%), *S*. *pneumoniae 35* (8.6%)*, and H*. *influenzae* (27) (6.6%) (Table 4).

In bivariate logistic regression analysis, age ranged 10–14 years (COR = 1.446, 95% CI: 0.954–2.193, *p* = 0.082); no formal education of guardians (COR = 2.369, 95% CI: 0.952–5.898, *p* = 0.064); primary education of guardians (COR = 1.730, 95% CI: 0.721–4.149, *p* = 0.219); household monthly income up to 1050 ETB (COR = 2.516, 95% CI: 1.306–4.848, *p* = 0.006); between 1051 and 3342 ETB (COR = 1.791, 95% CI: 1.061–3.021, *p* = 0.029); total household size greater or equal to five (COR = 1.429, 95% CI: 0.918–2.225, *p* = 0.114); enlarged, tender glands (lymph nodes) in the neck (COR = 2.595, 95% CI: 1.692–3.980, *p* < 0.001); history of recurrent tonsillitis (> 3 time in a year) (COR = 1.835, 95% CI: 1.197–2.813, *p* < 0.005); history of tonsillitis in the family (COR = 1.459, 95% CI: 0.960–2.217, *p* < 0.077); and smokers in the household (COR = 1.819, 95% CI: 0.719–4.599, *p* < 0.206) were candidate variable for multivariate analysis with *p* value < 0.250.

However, in multivariate analysis, children who were born from families who earn below 1050 ETB average monthly income (AOR = 2.306, 95% CI: 1.149, 4.627) were more exposed to bacterial tonsillitis than those who had better monthly income. A child who had enlarged or tender glands (lymph nodes) in the neck was (AOR = 2.468, 95% CI: 1.571, 3.878) about 2.5 times more likely to be exposed to bacterial tonsillitis than their counterparts. In addition to this, children with a history of recurrent tonsillitis more than three times in a year (AOR = 1.854, 95% CI: 1.178, 2.918) were 1.9 times more likely to be exposed to bacterial tonsillitis than those who had no history of recurrent tonsillitis (Table 5).

### 3.4. Antimicrobial Susceptibility Patterns of Bacterial Isolates

#### 3.4.1. Gram-Positive Bacteria

In this study, Gram-positive bacteria were sensitive to ampicillin (76.0%), amoxicillin/clavulanic acid (184) (75.6%), azithromycin (195) (88.6%), ceftriaxone (175) (72.3%), erythromycin (144) (77.4%), and vancomycin (144) (77.4%), while they were resistant to penicillin (61) (32.8%), cephalexin (65) (31.0%), chloramphenicol (70) (28.9%), clindamycin (67) (27.7%), ceftriaxone (62) (25.6%), and tetracycline (47) (25.3%). *S. pyogenes* was sensitive to azithromycin (67) (95.7%), chloramphenicol (61) (87.1%), and vancomycin (60) (85.7%). *S. pyogenes* was resistant to cephalexin (32) (45.7%), penicillin (31) (44.3%), and ceftriaxone (30) (42.9%). *S. aureus* was sensitive to azithromycin (56) (94.9%), penicillin (50) (84.7%), and amoxicillin/clavulanic acid (48) (81.4%) (Table 6).

#### 3.4.2. Gram-Negative Bacteria

Gram-negative bacteria were sensitive to azithromycin (30) (88.2%), ceftriaxone (30) (88.2%), and amoxicillin/clavulanic acid (27) (79.4%). *H*. *influenzae* was sensitive to azithromycin (24) (88.9%), ceftriaxone (24) (88.9%), and ciprofloxacin (22) (81.5%). *H*. *influenzae* was resistant to chloramphenicol (11) (40.7%). Six (85.7%) of *Klebsiella pneumoniae* were sensitive to azithromycin, ceftriaxone, chloramphenicol, and ciprofloxacin (Table 7).

### 3.5. Multiple Drug Resistance (MDR) Patterns of the Isolates

In this study, the overall p of MDR was 89/276 (32.25%), of which 86 (31.2%) were Gram-positive and three (1.1%) were shared by Gram-negative bacterial isolates. The most drug-R isolate was *S. pneumoniae* (33) (47.1%), followed by *S. pyogenes* and *S. aureus* (21) (35.6%). A majority of drug-R bacterial isolates were categorized as R to three classes of antibiotics (54) (19.6%) (Table 8).

## 4. Discussion

In this study, the overall p of bacterial tonsillitis among children with tonsillitis was 276 (67.6%). This study's finding was comparable to the result reported from India (70%) [10]. This study's finding was lower than the study in Iraq (89%) [11] and higher than the report in Egypt (61.4%) [12]. The variations in p could be attributed to differences in geographical locations, study designs, and population characteristics. This study identified a higher p of Gram-positive bacteria (242) (87.7%), consistent with previous findings from Egypt (84.7%) [13]. However, this rate was higher than that reported in India (57%) [10] but lower than the 100% p found in another Egyptian study [14]. The p of Gram-negative bacteria (34) (12.3%) was in agreement with a study in Egypt (15.3%) [15] but higher than a study done in Iraq (3%) [16] and lower than studies reported from India (38%) [10] and Korea (33.8%) [17]. These discrepancies may result from differences in diagnostic methods, sample types, and environmental factors.

The dominance of *S*. *pyogenes* (70) (25.4%) as the primary bacterial isolate was consistent with studies reported in Al-Jamhori Teaching Hospital, Iraq (25) (21.4%) [18]; India (66) (22.25%) [19]; and Nasiriyah, Iraq (34) (35.5%) [20]. Similarly, *S. aureus* was the second most common isolate (59) (21.4%), matching findings from Iraq (19.5%) [21]. However, it was higher than that reported in India (12.6%) [19], (13.7%) [22], Nigeria (12.83%), and Egypt (14.8%) [23], yet lower than some other studies conducted in Iraq (89.05%) [20], (50%) [11]. These variations could be due to differences in the bacterial strains circulating in different regions or the antibiotic usage patterns in those areas.

The proportion of the *S. epidermidis* was 56 (20.3%). This finding was higher than studies conducted in Iraq (8.4%) [21] (2.2%) [24]. The finding of *S. pneumoniae* was 35 (12.7%), which was in agreement with the findings from Egypt (11.4%) [25], (13.8%) [26], and India (13.95%) [27]. However, it was higher than another study conducted in Egypt (3.0%) [12]. The study's findings showed that *H. influenzae* was 27 (9.8%). This result was consistent with the study in India (6.7%) [22] and lower than the study conducted in Iraq (46.6%) [24] and Korea (23.4%) [28] and higher than the study in Pakistan (3.1%) [16]. Variations in magnitude and bacterial profile may be due to geographical location, study period, study design, socioeconomic status of the study population, and type of sample.

In this study, *S. pyogenes* was highly sensitive to azithromycin (95.7%), chloramphenicol (87.1%), and vancomycin (85.7%), and *S. aureus* was highly sensitive to azithromycin (94.9%), penicillin (84.7%), amoxicillin/clavulanic acid (81.4%), and ceftriaxone (80.4%). Consistent results were reported in Nigeria that *S. pyogenes* and *S. aureus* were sensitive to azithromycin (100%) [29]. In Iraqi, *S. pyogenes* was sensitive to vancomycin (81.9%) and erythromycin (81.4%), and *S. aureus* was sensitive to vancomycin (80%) and erythromycin (80%) [30]. In Brazil, *S. aureus* was susceptible to amoxicillin/clavulanic acid (86.1%) [31]. In Yemen, *S. pyogenes* was sensitive to azithromycin (100%) [18].

Unlike this finding, a study in Egypt showed *S. aureus* was less sensitive to erythromycin (39.5%) and vancomycin (32.9%) [32], and a study in Nigeria showed *S. pyogenes* was less sensitive to azithromycin (60%), amoxicillin/clavulanic acid (38.5%), and ampicillin (15.4%), and *S. aureus* was also less sensitive to azithromycin (45.5%), amoxicillin/clavulanic acid (27.3%), and ampicillin (36.3%) [33]. Another study in Egypt showed *S. pyogenes* was less sensitive to amoxicillin/clavulanic acid (20%) and not sensitive to erythromycin (0%) [34].

The current study showed S. *pyogenes* was less sensitive to penicillin (54.3%), clindamycin (55.4%), ceftriaxone (54.3%), and ciprofloxacin (56.4%). These findings were similar to a study conducted in Egypt (cephalexin [62.5%], erythromycin [43.75%], and ciprofloxacin [50%]) [35] and in Benin (cephalexin [64.7%]) [36]. This study reported *S. aureus* was less sensitive to chloramphenicol (62.7%), consistent with a study conducted in Iraq [21].

S. *pneumonia* was highly susceptible to ampicillin (91.4%), amoxicillin/clavulanic acid (88.8%), and azithromycin (80%), which was in agreement with studies in Iraq (azithromycin [94.4%]) [18] and in Egypt (azithromycin [100%]) [35].

*H. influenzae* was highly sensitive to azithromycin (85.7%) and chloramphenicol (83.3%). The findings were concurrent with findings in Iraq (azithromycin [90%]) [18]. However, relatively lower sensitivity findings were reported in Nigeria (azithromycin [60%] and chloramphenicol [55%]) [37].

MDR status of the isolates was *S. pneumoniae* (51.4%), *S. pyogenes* (47.1%), *S. aureus* (35.6%), *Streptococcus* spp. (Viridans) (30.0%), and S. *agalactiae* (16.7%). Similarly, MDR isolates were reported in China [38], Nigeria [36], India [39], and Egypt [14]. Unlike the present finding, lower MDR *S. aureus* (18%) was reported in Brazil [40].

The present study showed children who were born from low monthly-income families were two times more likely to be exposed to bacterial tonsillitis than those who earned better monthly income. This was consistent with two studies in India [39, 41]. Furthermore, children who had enlarged or tender glands and a history of recurrent tonsillitis were more exposed to having bacterial tonsillitis than their counterparts. Similar reports were obtained in Tanzania [42] and Somalia [43]. These findings highlight the importance of addressing social determinants of health in managing and preventing bacterial tonsillitis.

## 5. Limitations of the Study

This study considered only pathogenic bacteria; other etiological agents were not addressed. A recall bias by study participants may exist. Seasonal variations were not considered in this study.

## 6. Conclusion

In the present study, the overall p of bacterial tonsillitis among children with tonsillitis at selected health facilities was high. The major identified bacteria were *S. pyogenes*, *S. aureus*, *S. epidermidis*, *S. pneumoniae*, and *H. influenzae*. The isolates showed resistance to common antibiotics such as penicillin, chloramphenicol, clindamycin, ceftriaxone, and cephalexin. Nearly half of *S. pyogenes* and *S. pneumoniae* were MDR. Children who were born from low monthly-income families with enlarged or tender glands and a history of recurrent tonsillitis were more exposed to bacterial tonsillitis than their counterparts. Therefore, conducting culture and drug susceptibility tests is essential for effectively managing bacterial tonsillitis in the study area.

## Figures and Tables

**Table 1:** Sociodemographic characteristics among children with tonsillitis from selected health facilities in Hawassa City, 2021 (*n* = 408).

**Variable**	**Frequency**	**Percent (%)**
Sex		
Male	175	42.9
Female	233	57.1
Age		
5–9 years	217	53.2
10–14 years	191	46.8
Residence		
Urban	347	85.0
Rural	61	15.0
Guardian educational status		
No formal education	33	8.1
Elementary	30	7.4
High school	192	47.1
College/university	153	37.5
Household monthly income		
1050 ETB	90	22.1
1051–3342 ETB	240	58.8
> 3342 ETB	78	19.1
Family size		
< 5	284	69.6
≥ 5	124	30.4

Abbreviation: ETB, Ethiopian birr (unit of currency).

**Table 2:** Behavioral and clinical background of children with tonsillitis from selected health facilities in Hawassa City, 2021 (*n* = 408).

**Variables**	**Frequency**	**Percent (%)**
History of recurrent tonsillitis		
Yes	226	55.4
No	182	44.6
History of tonsillitis in the family		
No	209	51.2
Yes	199	48.8
Cigarette smokers in the household		
No	380	93.1
Yes	28	6.9
Spent day time		
Home	149	36.5
School	259	63.5
Live in a clean environment		
Yes	210	51.5
No	198	48.5
Eat spicy foods		
Yes	232	56.9
No	176	43.1
Longer hospital stay		
Yes	179	43.9
No	229	56.1
Tonsillitis while eating or drinking cold food		
Yes	202	49.5
No	206	50.5

**Table 3:** Signs and symptoms of the children with tonsillitis at selected health facilities in Hawassa City, 2021 (*n* = 408).

**Variables**	**Frequency**	**Percent (%)**
Fever		
Yes	353	86.5
No	55	13.5
White or yellow coating or patches on the tonsils		
Yes	286	70.1
No	122	29.9
A scratchy, muffled/throaty voice		
Yes	285	69.9
No	123	30.1
Sore throat		
Yes	280	68.6
No	128	31.4
Difficult/painful swallowing		
Yes	275	67.4
No	133	32.6
Headache		
Yes	263	64.5
No	145	35.5
Bad breath		
Yes	257	63.0
No	151	37.0
Red, swollen tonsils		
Yes	253	62.0
No	155	38.0
Enlarged, tender glands		
Yes	250	61.3
No	158	38.7
Exudate		
Yes	230	56.4
No	178	43.6

**Table 4:** Prevalence of bacterial species isolated from the children with tonsillitis at selected health facilities in Hawassa City, 2021 (*n* = 276).

**Variables**	**Frequency**	**Percent (%)**
*S. pyogenes*	70	17.2
*S. aureus*	59	14.5
*S. epidermidis*	56	13.7
*S. pneumoniae*	35	8.6
*H. influenzae*	27	6.6
*S. agalactiae*	12	2.9
*Streptococcus* spp. (Viridans)	10	2.5
*K. pneumoniae*	7	1.7
No bacterial growth	132	32.4

**Table 5:** Bivariate and multivariable logistic regression analysis for bacterial tonsillitis among children at selected health facilities in Hawassa City, 2021 (*n* = 408).

**Variables**	**Bacterial growth**		**COR (95% CI)**	**p** ** value**	**AOR (95% CI)**	**p** ** value**
	**Yes (%)**	**No (%)**				
Age (years)						
5–9	155 (56.2)	62 (47.0)	1		1	
10–14	121 (43.8)	70 (53.0)	1.446 (0.954, 2.193)	0.082	1.480 (0.951, 2.301)	0.082
Sex						
Female	118 (42.8)	57 (43.2)	1			
Male	158 (57.2)	75 (56.8)	1.018 (0.669, 1.547)	0.935		
Residence						
Urban	38 (13.8)	23 (17.4)	1			
Rural	238 (86.2)	109 (82.6)	1.322 (0.751, 2.326)	0.334		
Guardian education						
No formal education	27 (9.8)	6 (4.5)	2.369 (0.952, 5.898)	0.064	1.851 (0.701, 4.885)	0.214
Primary	23 (8.3)	7 (5.3)	1.730 (0.721, 4.149)	0.219	1.392 (0.541, 3.586)	0.493
Secondary and above	226 (81.9)	119 (90.2)	1		1	
Household monthly income						
1050 ETB	68 (24.6)	22 (16.7)	2.516 (1.306, 4.848)	0.006	2.306 (1.149, 4.627)	0.019
1051–3342 ETB	165 (59.8)	75 (56.8)	1.791 (1.061, 3.021)	0.029	1.726 (0.992, 3.003)	0.054
> 3342 ETB	43 (15.6)	35 (26.5)	1		1	
Family size						
< 5	199 (72.1)	85 (64.4)	1		1	
≥ 5	77 (27.9)	47 (35.6)	1.429 (0.918, 2.225)	0.114	1.422 (0.883, 2.291)	0.147
Enlarged, tender glands in the neck						
No	109 (39.5)	83 (62.9)	1		1	
Yes	167 (60.5)	49 (37.1)	2.595 (1.692, 3.980)	< 0.001	2.468 (1.571, 3.878)	< 0.001
History of recurrent tonsillitis						
No	137 (49.6)	85 (64.4)	1		1	
Yes	139 (50.4)	47 (35.6)	1.835 (1.197, 2.813)	0.005	1.854 (1.178, 2.918)	0.008
History of tonsillitis in the family						
No	133 (48.2)	76 (57.6)	1		1	
Yes	143 (51.8)	56 (42.4)	1.459 (0.960, 2.217)	0.077	1.307 (0.836, 2.043)	0.241
Cigarette smokers in the household						
No	254 (92.0)	126 (95.5)	1		1	
Yes	22 (8.0)	6 (4.5)	1.819 (0.719, 4.599)	0.206	1.003 (0.367, 2.737)	0.996
Spend day time						
Home	102 (37.0)	47 (35.6)	1			
School	174 (63.0)	85 (64.4)	1.060 (0.688, 1.633)	0.791		
Live in a clean environment						
No	130 (47.1)	68 (51.5)	1.193 (0.788, 1.807)	0.404		
Yes	146 (52.9)	64 (48.5)	1			
Consume spicy foods						
No	116 (42.0)	60 (45.5)	1			
Yes	160 (58.0)	72 (54.5)	1.149 (0.757, 1.745)	0.513		
Longer hospital stay						
No	150 (54.3)	79 (59.8)	1			
Yes	126 (45.7)	53 (40.2)	1.252 (0.822, 1.907)	0.295		
Tonsillitis while eating/drinking cold food						
No	135 (48.9)	71 (53.8)	1			
Yes	141 (51.1)	61 (46.2)	1.216 (0.802, 1.842)	0.357		

Abbreviations: AOR, adjusted odds ratio; CI, confidence interval; COR, crude odds ratio.

**Table 6:** Antibiotic susceptibility patterns of Gram-positive bacterial isolates from children with tonsillitis at selected health facilities in Hawassa City, 2021 (*n* = 242).

**Isolates**	**Pattern**	**Antibiotics (frequency [%])**										
		**AMP**	**AMC**	**AZT**	**CEP**	**CEF**	**CAF**	**CLD**	**ERH**	**TTC**	**PEN**	**VAC**
*S. pyogenes* (*N* = 70)	S	50 (71.4)	45 (64.3)	67 (95.7)	36 (51.4)	38 (54.3)	61 (87.1)	39 (55.7)	49 (70.0)	56 (80.0)	38 (54.3)	60 (85.7)
	I	0 (0.0)	0 (0.0)	0 (0.0)	2 (2.9)	2 (2.9)	1 (1.4)	9 (12.9)	3 (4.3)	1 (1.4)	1 (1.4)	1 (1.4)
	R	20 (28.6)	25 (35.7)	3 (4.3)	32 (45.7)	30 (42.9)	8 (11.4)	22 (31.4)	18 (25.7)	13 (18.6)	31 (44.3)	9 (12.9)

*S. aureus* (*N* = 59)	S	46 (78.0)	48 (81.4)	56 (94.9)	40 (67.8)	48 (80.4)	37 (62.7)	41 (69.5)	46 (78.0)	48 (76.3)	50 (84.7)	44 (74.6)
	I	1 (1.7)	1 (1.7)	1 (1.7)	4 (6.8)	0 (0.0)	0 (0.0)	1 (1.7)	0 (0.0)	0 (0.0)	1 (1.4)	0 (0.0)
	R	12 (20.3)	10 (16.9)	2 (3.4)	15 (25.9)	11 (18.6)	22 (37.3)	17 (28.8)	13 (22.0)	14 (23.7)	8 (13.6)	15 (25.4)

*S. epidermidis* (*N* = 56)	S	40 (71.4)	43 (76.8)	44 (78.6)	34 (60.7)	45 (80.4)	36 (64.3)	46 (82.1)	NR	NR	NR	NR
	I	1 (1.8)	0 (0.0)	3 (5.4)	7 (12.5)	1 (1.8)	1 (1.8)	0 (0.0)				
	R	15 (26.8)	13 (23.2)	9 (16.1)	15 (26.8)	10 (17.9)	19 (33.9)	10 (17.9)				

*S. pneumoniae* (*N* = 35)	S	32 (91.4)	31 (88.6)	28 (80.0)	18 (72.0)	26 (74.3)	20 (57.1)	22 (62.9)	30 (85.7)	23 (65.7)	16 (45.7)	22 (62.9)
	I	0 (00.0)	1 (2.9)	2 (5.7)	4 (16.0)	1 (1.8)	0 (0.0)	0 (0.0)	1 (2.9)	0 (0.0)	2 (20.0)	0 (0.0)
	R	3 (8.6)	3 (8.6)	5 (14.3)	3 (12.0)	8 (22.9)	15 (42.9)	13 (37.1)	4 (11.4)	12 (34.3)	17 (48.6)	13 (37.1)

*S. agalactiae* (*N* = 12)	S	8 (66.7)	8 (66.7)	NR	NR	10 (83.3)	10 (83.3)	8 (66.7)	10 (83.3)	8 (66.7)	9 (75.0)	11 (91.7)
	I	2 (16.7)	0 (0.0)			0 (0.0)	0 (0.0)	3 (25)	1 (8.3)	0 (8.0)	1 (8.3)	0 (0.0)
	R	2 (16.7)	4 (33.3)			2 (16.7)	2 (16.7)	1 (8.3)	1 (8.3)	4 (33.3)	2 (16.7)	1 (8.3)

*Streptococcus* spp. (Viridans) (*N* = 10)	S	8 (80.0)	8 (80.0)	NR	NR	8 (80.0)	6 (60.0)	3 (30.0)	9 (90.0)	6 (60.0)	3 (30.0)	7 (70.0)
	I	0 (00.0)	1 (10.0)			1 (10.0)	0 (00.0)	3 (30.0)	0 (0.0)	1 (10.0)	4 (40.0)	0 (0.0)
	R	2 (20.0)	1 (10.0)			1 (10.0)	4 (40.0)	4 (40.0)	1 (10.0)	3 (30.0)	3 (30.0)	3 (30.0)

Total (*N* = 242)	S	184 (76.0)	183 (75.6)	195 (88.6)	128 (60.1)	175 (72.3)	170 (70.4)	159 (65.7)	144 (77.4)	141 (74.6)	116 (62.4)	144 (77.4)
	I	4 (1.7)	3 (1.3)	6 (2.72)	17 (8.1)	5 (2.0)	2 (0.8)	16 (6.6)	5 (2.6)	1 (0.5)	9 (5.4)	1 (0.54)
	R	54 (22.3)	56 (23.1)	19 (8.6)	65 (31.0)	62 (25.6)	70 (28.9)	67 (27.7)	37 (19.9)	47 (25.3)	61 (32.8)	41 (22.0)

Abbreviations: AMC, amoxicillin/clavulanic acid; AMP, ampicillin; AZT, azithromycin; CAF, chloramphenicol; CEF, ceftriaxone; CEP, cephalexin; CLD, clindamycin; ERH, erythromycin; I, intermediate; NR, not recommended; PEN, penicillin; R, resistance; S, sensitivity; TTC, tetracycline; VAC, vancomycin.

**Table 7:** Antibiotic susceptibility patterns of both Gram-negative isolates among children with tonsillitis at selected health facilities in Hawassa town, 2021 (*n* = 34).

**Isolates**	**Pattern**	**Antibiotics (frequency [%])**						
		**AMP**	**AUG**	**AZT**	**CEP**	**CEF**	**CAF**	**CPR**
*H. influenzae* (*N* = 27)	S	21 (7.8)	22 (81.5)	24 (88.9)	NR	24 (88.9)	12 (44.4)	22 (81.5)
	I	0 (0.0)	1 (3.7)	0 (0.0)		0 (0.0)	4 (14.8)	0 (0.0)
	R	6 (22.2)	4 (14.8)	3 (11.1)		3 (11.1)	11 (40.7)	5 (18.5)

*K. pneumoniae* (*N* = 7)	S	NR	5 (71.4)	6 (85.7)	0 (0.0)	6 (85.7)	6 (85.7)	6 (85.7)
	I		0 (0.0)	0 (0.0)	6 (85.7)	0 (0.0)	0 (0.0)	0 (0.0)
	R		2 (28.6)	1 (14.3)	1 (14.3)	1 (14.3)	1 (14.3)	1 (14.3)

Total (*N* = 34)	S	21 (66.7)	27 (79.4)	30 (88.2)	0 (0.0)	30 (88.2)	18 (53.0)	28 (78.6)
	I	0 (11.1)	1 (2.94)	0 (0.0)	6 (85.7)	0 (0.0)	4 (11.76)	0 (0.0)
	R	6 (22.2)	6 (17.6)	4 (11.8)	1 (14.3)	4 (11.8)	12 (35.3)	6 (21.4)

Abbreviations: AMP, ampicillin; AUG, amoxicillin–augmentin; AZT, azithromycin; CAF, chloramphenicol; CEF, ceftriaxone; CEP, cephalexin; CLD, clindamycin; CPR, ciprofloxacin; ERH, erythromycin; I, intermediate; NR, not recommended; PEN, penicillin; R, resistance; S, sensitivity; TTC, tetracycline; VAC, vancomycin.

**Table 8:** Multidrug-resistant rates of bacterial isolates among children with tonsillitis at selected health facilities in Hawassa town, 2021.

**Bacterial isolates**	**n** ** (%)**	**R0 (%)**	**R1 (%)**	**R2 (%)**	**R3 (%)**	**R4 (%)**	**R5 (%)**	**≥ R6 (%)**	**MDR (** **n** ** [%])**
*S. pyogenes*	70	7 (10.0)	20 (28.6)	10 (14.3)	23 (32.9)	5 (7.1)	3 (4.3)	2 (2.9)	33 (47.1)
*S. aureus*	59	16 (27.1)	16 (27.1)	6 (10.2)	11 (18.6)	5 (8.5)	1 (1.7)	4 (6.8)	21 (35.6)
*S. epidermidis*	56	15 (26.8)	19 (33.9)	13 (23.2)	5 (8.9)	4 (7.1)	0 (0.0)	0 (0.0)	9 (16.1)
*S. pneumoniae*	35	2 (5.7)	8 (22.9)	7 (20.0)	10 (28.6)	3 (8.6)	1 (2.9)	4 (11.4)	18 (51.4)
*H. influenzae*	27	13 (48.1)	7 (25.9)	4 (14.8)	3 (11.1)	0 (0.0)	0 (0.0)	0 (0.0)	3 (11.1)
*S. agalactiae*	12	2 (16.7)	4 (33.3)	4 (33.3)	1 (8.3)	1 (8.3)	0 (0.0)	0 (0.0)	2 (16.7)
*Streptococcus* spp. (Viridans)	10	0 (0.0)	3 (30.0)	4 (40.0)	1 (10.0)	1 (10.0)	1 (10.0)	0 (0.0)	3 (30.0)
*K. pneumoniae*	7	3 (42.9)	2 (28.6)	2 (28.6)	0 (0.0)	0 (0.0)	0 (0.0)	0 (0.0)	0 (0.0)
Total	276	58 (21.0)	79 (28.6)	50 (18.1)	54 (19.6)	19 (6.9)	6 (2.1)	10 (3.6)	89/276 (32.24%)

*Note:* MDR, resistance to three or more antibiotics; R0, no resistance to tested antibiotics; R1, resistance to one antibiotic; R2, resistance to two antibiotics; R3, resistance to three antibiotics; R4, resistance to four antibiotics; R5, resistance to five antibiotics; ≥ R6, resistance to six and more than six antibiotics.

Abbreviations: R, resistance; *n*, number.

## Data Availability

The datasets used and/or analyzed during the study are available from the corresponding author upon reasonable request.

## Nomenclature

AST: antimicrobial susceptibility test
GABHS: Group A beta-hemolytic streptococci
MDR: multiple drug resistance
OPD: outpatient department
